# The Potential and Challenges of Exploiting the Vast But Dynamic Neoepitope Landscape for Immunotherapy

**DOI:** 10.3389/fimmu.2017.01113

**Published:** 2017-09-11

**Authors:** Els M. E. Verdegaal, Sjoerd H. van der Burg

**Affiliations:** ^1^Experimental Cancer Immunology and Therapy Group, Leiden University Medical Center, Department of Medical Oncology, Leiden, Netherlands

**Keywords:** somatic mutations, neoepitopes, immunotherapy, tumor heterogeneity, vaccination, adoptive cell therapy

## Abstract

Somatic non-synonymous mutations in the DNA of tumor cells may result in the presentation of tumor-specific peptides to T cells. The recognition of these so-called neoepitopes now has been firmly linked to the clinical success of checkpoint blockade and adoptive T cell therapy. Following proof-of-principle studies in preclinical models there was a surge of strategies to identify and exploit genetically defined clonally expressed neoepitopes. These approaches assume that neoepitope availability remains stable during tumor progression but tumor genetics has taught us otherwise. Under the pressure of the immune system, neoepitope expression dynamically evolves rendering neoepitope specific T cells ineffective. This implies that the immunotherapeutic strategy applied should be flexible in order to cope with these changes and/or aiming at a broad range of epitopes to prevent the development of escape variants. Here, we will address the heterogeneous and dynamic expression of neoepitopes and describe our perspective and demonstrate possibilities how to further exploit the clinical potential of the neoepitope repertoire.

## Introduction

Spectacular progress has been made in the treatment of cancer by the introduction of checkpoint blocking antibodies against the inhibitory molecules CTLA-4, and PD-1 or its ligand PD-L1 (1, 2). The efficacy of these antibodies depends on the presence of antigen-specific T cells that can recognize tumor cells but are functionally inhibited in cancer patients (3). In melanoma (4) and lung cancer (5), clinical benefit of checkpoint blocking therapy strongly correlates with the presence of a high mutational load. This led to the hypothesis that a high number of somatic non-synonymous mutations may result in the formation of so-called neoepitopes that are recognized as truly foreign by CD4^+^ and CD8^+^ T cells, the response of which is unleashed by checkpoint blocking.

**Figure 1:**
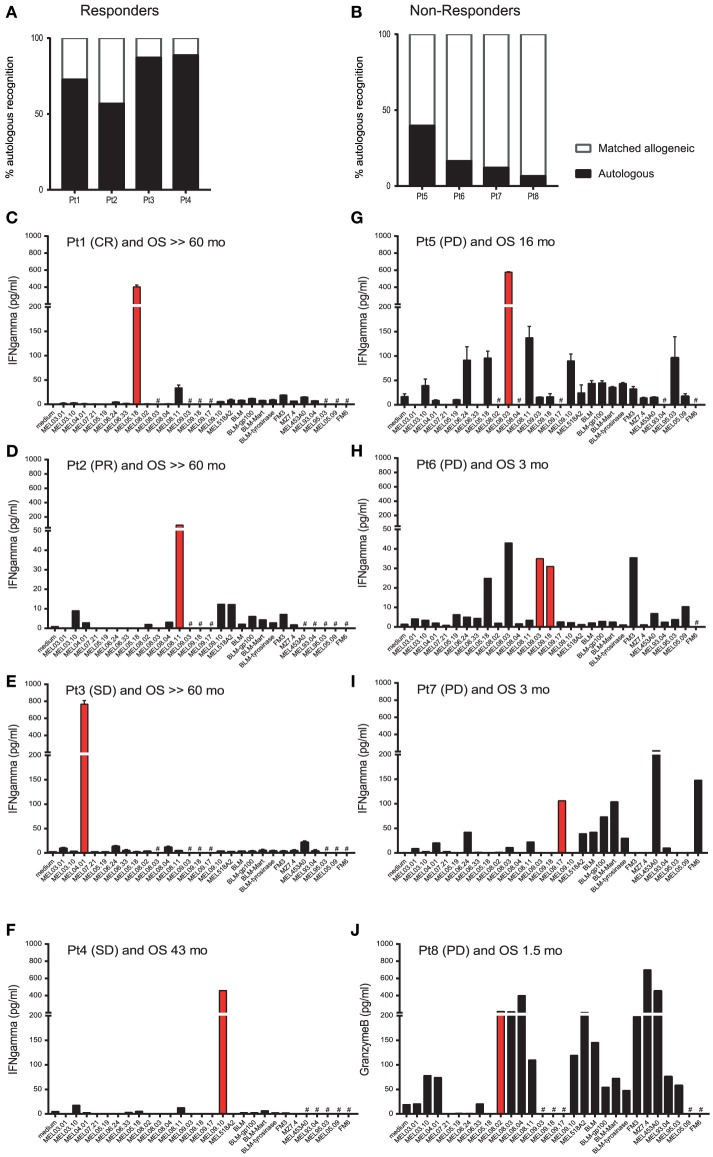
T cell batches administered to responder patients recognize private rather than shared antigens. Tumor-reactive T cell batches were generated by repeated stimulation of PBMC with autologous melanoma cell lines in a mixed lymphocyte tumor cell culture (MLTC). These T cells were administered to melanoma patients by ACT. The patient number, with best overall response [complete response (CR), partial response (PR), stable disease (SD), or progressive disease (PD)], and overall survival (OS) in months are given. (# = not done). IFN-gamma production, as an indicator of T cell activation, was measured after incubation of T cells used for ACT with various (partially-)matched HLA class-I melanoma cell lines. The IFN-gamma production of T cells against the autologous tumor cells is depicted as a fraction of the total IFN-gamma production against all tested cells (set at 100%) for responder patients (*n* = 4) and non-responder patients (*n* = 4) in panels **(A,B)**, respectively. The data of each individual patient are given in panels **(C–J)**. Data in panels **(C,D)** were previously reported (11). IFN-gamma production upon recognition of each cell line is represented by separate bars. The red bar in each panel indicates the autologous melanoma cell line that was used to generate the corresponding T cell batch. The patients were treated in a clinical trial approved by the local ethics committee (LUMC study P04.085) and all patients gave written informed consent.

It was suggested that the clinical efficacy of adoptive cell therapy (ACT) also relies on the presence of mutation-specific T cells in the infusion product. Indeed, tumor infiltrating T cell (TIL) used for successful ACT treatment of melanoma patients (6–11), head and neck cancer (12), cholangiocarcinoma (13, 14), and colorectal cancer (15) were shown to contain considerable frequencies of neoepitope-specific T cells. Furthermore, durable clinical responses were obtained when PBMC-derived tumor-reactive T cells, comprising almost exclusively clonally expressed neoepitope-specific CD8^+^ and CD4^+^ T cells, were infused (10, 11, 16). Furthermore, we observed that ACT products administered to responder patients contained T cells that recognized private rather than shared antigens as demonstrated by their almost exclusive recognition of autologous tumor cells and not a series of HLA-matched melanoma cells (Figure 1). In contrast, T cells administered to non-responders showed a broad recognition pattern. Moreover, infusion of highly enriched neoepitope-specific T cells resulted in clear tumor regression in a patient who relapsed after bulk TIL therapy (13). Altogether, these data suggest that approaches to select, expand and activate neoepitope specific T cells by (combinations of) checkpoint blocking, ACT and/or vaccination can improve the clinical outcome of this treatment. This, however, does not mean that we should neglect the therapeutic potential of shared tumor-antigens. This is illustrated by the complete tumor eradication of melanoma after transfer of NY-ESO-1-specific CD4^+^ T cells (17) and genetically engineered NY-ESO-1 specific T cells (18, 19). Although shared tumor antigens are important targets for development of immunotherapy this review focuses exclusively on the exploitation of neoepitopes.

## Heterogeneity and Dynamics of Neoepitope Landscape

Optimal exploitation of neoepitope immunity for cancer therapy requires a thorough understanding of the neoantigen landscape. Several studies have shown that the mutational landscape of a tumor is not cut into stone but dynamically evolves (20–27) with as potential outcome that tumor recognition by the immune system is lost due to reduced or lost expression of neoepitopes in recurrent tumor cell clones (11). Hence, it not only is essential to gain knowledge of the frequency and extent of intratumoral heterogeneity but also of mutational landscape changes during tumor progression and regression after treatment, including immunotherapy.

Heterogeneity of mutations occurs at spatial and temporal levels. First, different areas within a single tumor lesion may harbor different mutations. In individual tumors of eight melanoma patients, the proportion of heterogeneity of somatic mutations was reported to range from 3 to 38%, although it should be mentioned that heterogeneity was particularly abundant in non-expressed genes. Nonetheless, a high degree of heterogeneity was associated with a more aggressive course of the disease (25). Second, mutations may differ between primary and metastatic lesions as well as between various metastases. Analysis of primary breast cancer lesions and matched metastases revealed that the number of genetic alterations was reduced in metastatic lesions. Although this might seem counterintuitive at first glance, it can be explained by a high grade of heterogeneous variants in the primary tumor, from which specific subclones with a less heterogeneous mutation expression but increased proliferative and metastatic potential evolve (20). Indeed, some of the mutations shared between metastatic lesions of various patients are linked to poor survival. The changes in the landscape of expressed (non-silenced) mutations vary depending on the cancer type illustrating that proportionally intratumoral heterogeneity is very high in glioma and low in NSCLC and melanoma [reviewed in Ref. (28)]. However, given the relatively high mutation rate in the latter two tumor types, the absolute number of alterations in expressed mutations is still high (28). In one exceptional case of a NSCLC patient, 99% of the total genetic alterations (point mutations, insertions, and deletions) differed between sequential lesions (24). Finally, mutations may vary between early lesions that are sensitive to treatment and treatment-resistant recurrences. The extent of genetic alterations in these recurrent lesions varies across cancer types and is very low in ovarian cancer (21, 22). At the other end of the spectrum are low-grade gliomas that acquire thousands of somatic mutations that differ from the initial lesions after temozolomide therapy (23) and concomitantly evolve into a high-grade glioma phenotype. Anagnostou et al. elegantly showed that tumor lesions recurring after checkpoint blocking therapy displayed both loss and gain of putative (mutation associated) neoepitopes in four NSCLC and one HNSC patients (27). We analyzed the expression stability of six clonally expressed T cell targeted neoepitopes in serially obtained tumors from two stage IV melanoma patients treated by ACT (11). The data from these paired tumor samples demonstrated that under the attack of T cells neoepitope availability was lost in four out of six cases in tumor subclones that evolved upon disease progression. These two studies show that immune pressure sculpts the mutational landscape of tumors and imply that flexibility toward the neoepitopes targeted is a prerequisite for immunotherapeutic approaches aiming to exploit the neoantigen repertoire. Recently it was reported that the number of recognized neoepitopes in TIL used for ACT of melanoma patients do not directly correlate to treatment outcome (29). There are several reasons to explain this, including the copresence of clinically active T cells reactive to tumor-associated antigens (17), which may have had a major contribution in the clinical responses obtained in patients who received TIL with low neoepitope-reactivity. In those patients who do not show clinical response after transfer of TIL with a high frequency of neoepitope-specific cells, a multitude of factors defined as the immunophenoscore (30, 31), including TME phenotype and tumor escape status, may have hampered clinical effectiveness.

Dynamics in neoantigen expression predict that strategies applying neoepitopes for reinforcement of antitumor immunity should aim at a broad panel of antigens in order to prevent escape variants. Hence, when an immunotherapeutic strategy requires epitope selection the highest priority should be given to neoepitopes derived from driver mutations. These mutations are expected to be expressed in the majority of—if not all—tumor cells and will not be lost by immunoediting because they are essential for the malignant phenotype. However, T cells reactive against these epitopes are infrequently detected even though several driver mutations are frequently present in various tumor types, including colorectal cancer and melanoma (15, 32, 33). Emphasis should also be given to neoepitopes derived from clonally expressed mutated genes other than acquired early during tumor evolution. In contrast to subclonal mutations, these clonal mutations may comprise driver and passenger mutations that are expressed in the “trunk” of the tumor evolutionary tree and therefore expressed in the majority of tumor cells. This notion is sustained by the observation that clinical benefit from checkpoint-blocking therapy is not only correlated with total tumor burden but also correlated with homogeneity of mutations within spatial and temporally different tumor lesions in NSCLC and melanoma patients (26).

## Exploiting the Potential of the Vast Number of Putative Neoepitopes

The correlation between the success of checkpoint blockade and the mutational load in NSCLC, melanoma and mismatch repair deficient tumors (4, 5, 34), demonstrates that metastasized late stage progressive cancers with concomitant high grade of intratumoral heterogeneity can be effectively targeted. It also underscores the adaptive capacity of the immune system to the dynamic mutational and neoepitope landscape. Similarly, we observed in a recurrent subclone after ACT that the expression of a non-targeted neoepitope was increased when compared to the earlier fully regressed lesions and this was paralleled by the emergence of intratumoral T cells specific for this neoepitope (11). However, still roughly halve of the patients do not respond to checkpoint-blocking therapy, part of which can be explained by a weak or absent pre-existing tumor-specific T cell response (3). Therefore, various therapeutic approaches aiming to enhance or induce (neo)antigen-specific T cell responses are pursued.

**Figure 2:**
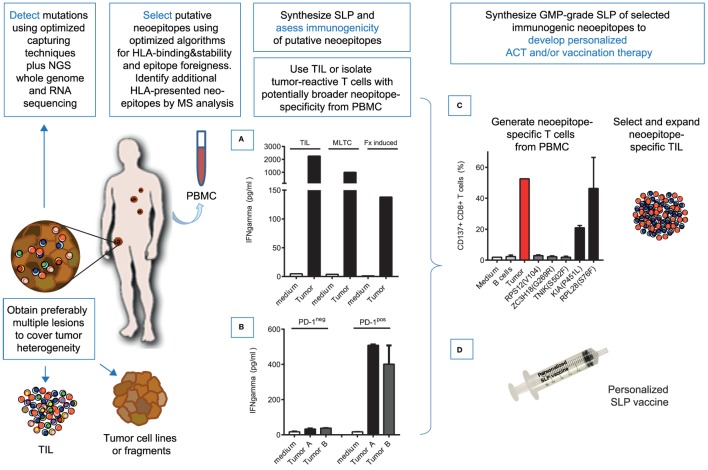
Proposed pipeline for individualized immunotherapy exploiting neoepitope-specific T cells. Tumor tissue is excised and used for: (1) whole-exome and RNA sequencing of tumor cells and matched normal cells using optimized capturing of DNA coding regions to identify somatic mutations in expressed genes. Preferably multiple lesions are used to minimize selection of mutations with heterogeneous or lost expression, (2) to establish a tumor cell line or prepare tumor fragments for generation of neoepitope-specific, tumor-reactive T cells, and (3) to culture tumor infiltrating T cells (TIL). Putative neoepitopes can be selected based on expression of the mutated gene. Further prioritization using optimized processing, MHC-binding and stability algorithms is optional but not essential. Next, synthetic long peptides (SLPs) harboring the selected putative neoepitopes are used to assess immunogenicity using TIL. As an alternative, tumor-reactive T cells obtained by repeated stimulation of PBMC with an autologous tumor cell line (MLTC) or small tumor fragments (Fx induced), exemplified in **(A)** or PD-1 positive cells selected from PBMC, exemplified in **(B)** that are shown to contain a potentially broader repertoire of neoepitope-specific, tumor-reactive T cells can be used. Identification of immunogenic neoepitopes is assessed by IFN-gamma production of tumor-reactive T cells upon coincubation of SLP-loaded autologous B cells as APC. This approach allows identification of CD4^+^ as well as CD8^+^ epitopes (10). Subsequently, selected immunogenic neoepitopes that are shown to elicit a T cell response, can be used to select specific T cells from PBMC or TIL for ACT **(C)** or for personalized vaccination **(D)**. **(C)** shows that neoepitope-specific T cells can be obtained from patient’s own PBMC by repeated peptide stimulation. PBMC were stimulated at day 0 with SLP harboring selected neoepitopes and stimulated at week 2 with PHA-blasts loaded with the corresponding short minimal CD8^+^ epitopes. After 4 weeks, the majority of the obtained cells were CD8^+^ T cells that recognized autologous B cells loaded with the specific SLP, as well as autologous tumor cells. Thus obtained enriched neoepitope- and tumor-reactive T cells can be expanded and used for ACT. Alternatively or in combination with ACT, prevalent neoepitope-specific T cells can be boosted by vaccination with SLP harboring the selected neoepitopes plus an immunostimulatory adjuvant to induce a robust immune response and/or to further support transferred neoepitope-specific T cells *in vivo*.

A logical option to harness the immune system is by identification and targeting of additional neoepitopes. So far, the number of neoepitopes eliciting a T cell response that are identified ranges from one to maximally ten per patient (35) and detection of neoepitope-specific T cells in ACT products or TILs has revealed that only a minority of the putative neoepitopes predicted to bind to HLA elicits spontaneous immune responses (7, 8, 10, 11, 14, 36, 37). The underlying reasons are yet unknown. Most likely the selection of mutated antigens for neoepitope identification based on NGS and RNA sequencing, the prediction algorithms and T cell tests are far from optimal and may be improved. For instance, by more efficient capture of coding DNA regions and/or comprehensive transcriptional analysis as well as by optimization of algorithms that predict peptide processing, HLA binding, HLA-peptide stability and peptide foreignness (38, 39), but also by improving T-cell detection methods. A sensitive and rapid identification method to identify functional immunogenic neoepitopes is the use of DNA barcoded MHC-multimers. This allows screening of a large number of peptides in a relatively small sample of PBMC, TIL or tumor-reactive T cells (40). The sensitivity of detection may be even further enhanced when proliferation of neoepitope-specific T cells is assessed by TCR Vbeta-clonality analysis of PBMC/TIL before and after *in vitro* stimulation (27). The frequency of neoepitope-specific T cells may be low and may therefore limit detection of neoepitope immunogenicity. Selection of tumor-specific T cells from PBMC may be applied to improve outcome of functional immunogenicity tests. Actually, PD-1^+^ CD8^+^ and not the more abundant PD-1^−^ CD8^+^ T cells from peripheral blood [Figure 2B and (36)] and also from TIL (41) were shown to harbor tumor-reactive and neoepitope-specific T cells. Rapid identification of multiple neoepitopes per tumor sample could be readily achieved using PD1^+^ CD8^+^ selected TIL (42) isolated directly *ex vivo* from tumor samples. It would be of interest to also investigate PD-1^+^ CD8^+^ T cells from PBMC of the corresponding patients to see whether reactivity to a similar repertoire of neoepitopes is detected. Other reasons for a failure to detect more neoepitope specific T cells might be that spontaneously triggered neoepitope-specific T cells are not activated due to neoepitope heterogeneity and in particular neoepitope expression between tumor subclones (11) or because they have become exhausted or anergic (43) in TIL.

There is already some evidence that there are more neoepitopes processed and presented in the HLA molecules at the tumor cell surface than those that spontaneously raise neoepitope-specific T cell immunity. Stronen et al. showed that putative neoepitopes, not recognized by TILs, were able to trigger tumor-reactive T-cell reactivity in PBMC from healthy donors, arguing that a “neglected neoepitope repertoire” exists (37). This is also supported by the work of Carreno et al. showing that vaccination with neoepitopes that are not spontaneously recognized, does result in a putative neoepitope-specific T cell response in three patients with melanoma (44). Two out of seven selected immunogenic HLA-A*0201-restricted neoepitopes used for vaccination of one patient, could be detected by mass spectrometry analysis to be endogenously expressed, processed and presented by HLA on tumor cells (44) and the T cells directed against these epitopes specifically lysed tumor cells expressing these two neo-antigens but not other target cells.

To gain more insight in the number of attended and neglected neoepitopes that are actually presented by HLA at the tumor cell surface mass spectrometry can be utilized. Optimal identification of neoepitopes using this approach would ideally require access to (a) a substantial amount of tumor tissue or preferentially a tumor cell line that can be cultured up to the quantities required; (b) somatic mutation data derived from sequenced exomes and transcriptome; and (c) autologous T cells to confirm immunogenicity of the neoepitope and functional recognition (something for which also HLA-matched naïve T cells from healthy donors can be used) as well as to show the presence of a functional T-cell repertoire in the patient, which is crucial for ultimate immune responsiveness. Identification of tumor-specific T cell epitopes from a fraction of tumor tissue using mass spectrometry may be limited because of the amount of available starting material for representative detection of neoepitopes among the entire HLA-ligandome (45). Nevertheless, immunogenic neoepitopes have been identified directly from melanoma biopsies (46). Mass spectrometry/ligandome data were matched with NGS/transcriptome data for a total of five patients and led to identification of four immunogenic epitopes. In addition to the identified neoepitopes, many known and novel peptide ligands derived from tumor-associated antigens were identified, demonstrating the applicability of mass spectrometry/proteomics for broad MHC peptide ligand identification.

## Harnessing the Immune System with Neoepitope Specific T Cells

From the above mentioned data, it is expected that also clinical efficacy of ACT or vaccination can be enhanced by focusing on (clonally) expressed mutations-derived neoepitopes. Once a set of immunogenic neoepitopes has been identified it can be used to induce or increase the frequency of tumor-reactive T cells by vaccination using RNA (47), synthetic long peptides (SLPs) (48), or antigen-loaded DC (49–53). Clinical trials applying vaccination with neoepitope RNA or SLPs recently demonstrated feasibility and clinical effectiveness of neoepitope-based personalized immunotherapy (47, 48).

As an alternative to vaccination, selected neoepitopes can be used to expand neoepitope-specific T cells *in vitro* for use in ACT, for instance by stimulation of patients PBMC with SLPs covering the selected neoepitopes. We showed that SLP-stimulated T cells not only respond to neoepitope peptide-pulsed APC but also recognized autologous tumor cells, indicating that they recognize endogenously naturally presented neoepitopes (Figure 2C) and as such have clinical potential. In order to speed-up this process, one may also preselect PD-1 positive cells from PBMC [Figure 2B and (36)] either with or without prior stimulation with autologous tumor cells or stimulation with small tumor fragments in case no autologous tumor cell line is available.

## Conclusion and Perspective How to Exploit the Clinical Potential of the Neoepitope Repertoire

Based on the correlations between successful checkpoint therapy and mutational load as well as successful ACT and the presence of neoepitope-specific T cells, it is fair to assume that these neoepitope-specific T cells strongly contribute to the clinical effect. Clearly, the immunotherapy-mediated increased immunological pressure on the tumor in the end results in the outgrowth of tumor cell clones with downregulated or lost expression of the targeted epitopes. In most cases without direct consequences for the tumor cell itself as most of the targeted mutations are not directly involved in tumorigenesis. Importantly, the number of identified spontaneously recognized neoepitopes probably is only a fraction of the total repertoire of tumor-presented tumor-specific as well as tumor-associated antigens. To prevent neoepitope escape this broader repertoire of neoepitopes should be targeted. This, however, requires crucial improvements both with respect to the identification and the speed of the process itself. These approaches all rely on the successful identification of targetable neoepitopes, which will not be possible for all patients. In cases where no immunogenic epitopes can be identified using TIL, stimulation of PBMC with autologous tumor cells or tumor cell fragments in mixed lymphocyte tumor cell cultures (MLTCs) may result in generation of a T cell product enriched for tumor-reactive T cells probably comprising considerable frequencies of undefined but effective neoepitope-specific T cells (Figure 1) with a broader neoepitope specificity when compared with TIL [Figure 2 and (54)].

Assuming that a selection of immunogenic neoepitopes is available, the question remains how to optimally implicate them in effective treatment. In our opinion, the complexity of tumor biology will eventually require a combined approach to effectively combat the patient’s tumor. First of all, the patient must be harnessed with tumor-reactive T cells, which can be accomplished by vaccination targeting neoepitopes (Figure 2D) or adoptive transfer of neoepitope specific, tumor-reactive T cells (Figure 2C). In addition, radiation and chemotherapy could be applied to induce tumor cell apoptosis, which can be considered as *in vivo* whole tumor cell vaccination, boosting the endogenous T cell response and stimulating antigen spreading and on top of that may promote DC trafficking and T cell priming and trafficking to non-infiltrated “cold” tumors (55–59). Moreover, chemotherapy may normalize the generally suppressive myeloid cell subsets and/or enhance the influx of potent APCs and thereby improve response to therapy (60–63). Finally, checkpoint-blocking therapy should be provided to allow optimal effector cell function of the neoepitope-specific effector T cells at the tumor site.

## Author Contributions

EV and SB designed and wrote the article.

## Conflict of Interest Statement

The authors declare that the research was conducted in the absence of any commercial or financial relationships that could be construed as a potential conflict of interest.
